# Neurological impairment and malnutrition in children: The role of home enteral nutrition in real life

**DOI:** 10.3389/fnut.2023.1087603

**Published:** 2023-03-22

**Authors:** Antonella Diamanti, Teresa Capriati, Antonella Mosca, Chiara Maria Trovato, Francesca Laureti, Bianca Mazzoli, Giulia Bolasco, Tamara Caldaro, Francesco De Peppo, Susanna Staccioli, Raffaele Edo Papa, Antonella Cerchiari, Paola De Angelis, Giuseppe Maggiore

**Affiliations:** ^1^Hepatology, Gastroenterology and Nutrition Unit, Bambino Gesù Children Hospital, Istituti di Ricovero e Cura a Carattere Scientifico (IRCCS), Rome, Italy; ^2^Unit of Palidoro Pediatric Surgery, Department of Specialized Surgery, Bambino Gesù Children's Hospital, IRCCS, Rome, Italy; ^3^Department of Neurorehabilitation, Bambino Gesù Children's Hospital, Rome, Italy; ^4^Pediatrics Unit, University Department of Pediatrics, Bambino Gesù Children's Hospital, IRCCS, Rome, Italy

**Keywords:** neurological, enteral nutrition, malnutrition, gastro-esophageal reflux disease (GERD), multidisciplinary care

## Abstract

**Objective:**

Recent decades have brought an increased survival of children with Neurologic Impairment (NI) but malnutrition and digestive comorbidity remain important challenges to face. We designed the present study to assess the course of nutritional status following standardized Home Enteral Nutrition (HEN) program and to evaluate impact of changing mode of feeding, as a part of overall multidisciplinary management, on digestive co-morbidity as Gastro-Esophageal Reflux Disease (GERD), Oropharyngeal Dysphagia (OPD), constipation and airway aspiration.

**Methods:**

We performed a retrospective analysis on NI children entered into Institutional HEN program due to NI disorders between January 2011 and 2019. Demographic, anthropometric characteristics (BMI z-score and weight for age z-score) and symptoms (GERD, OPD constipation and airway aspiration) were collected at the enrolment and during the follow up.

**Results:**

We enrolled 402 patients (median age: 39 months); overall survival was 97%. Nutritional status was significantly improved by HEN; in particular growth profile significantly changed within the first 2 years following HEN beginning; GERD and airways aspirations decreased after HEN beginning. Constipation and OPD remained unchanged over time.

**Conclusions:**

Malnutrition and digestive complaints are distinctive features of NI children. Nutritional status improve after 2 years from the beginning of standardized nutritional interventions. Overall multidisciplinary care, including standardized HEN protocols, seems to also impact on GERD and airway aspirations, which can decrease over time. It is possible that constipation and OPD, unchanged over time, are more dependent on underlying diseases than on overall treatments.

## Introduction

Recent decades have brought increased survival of children with Neurologic Impairment (NI) but malnutrition, related to uneasy energy requirements assessment, severe Gastro-Esophageal Reflux Disease (GERD), swallowing disorders and feeding aversion, remains an important challenge to face in these children (1, 2). Appropriate timing and extent of nutritional interventions in NI children have been the focus of position statements and guidelines (2–4).

Enteral Nutrition (EN), started in hospital and continued at home as Home Enteral Nutrition (HEN), is one of the first steps of the multidisciplinary management of NI children (1, 4, 5) and it should be continuously re-modulated according to growth and co-morbidity.

Over the last decades, in our Institution, several patients with NI had a comprehensive rehabilitative program that included continuous surveillance of nutritional status and co-morbidity by the multidisciplinary team.

Therefore, aims of this study are to assess the course of nutritional status following a standardized HEN program and to evaluate the impact of changing mode of feeding, as a part of overall multidisciplinary management, on digestive co-morbidity [GERD, Oropharyngeal Dysphagia (OPD), constipation and airway aspiration].

## 2. Patients and methods

### 2.1. Patients

We performed a retrospective analysis on all NI children (9 months−18 years) admitted for the first time into the Institutional HEN program between January 2011–2019 and with at least 2 years of follow-up. Children with Progressive Neurologic Impairment (PNI); such as metabolic encephalopathies, neuromuscular diseases, leukoencephalopathy, genetic encephalopathy, malformative enecephalopathy, West syndrome and Rett syndrome, and children with Non-Progressive Neurologic Impairment (NPNI), such as infantile cerebral palsy were enrolled. All patients were followed up and cared for by a multidisciplinary team of “Bambino Gesù” Children's Hospital. We excluded patients starting HEN before first admitted at our unit, NI patients in parenteral nutrition and with other gastrointestinal disorders (short bowel syndrome, celiac disease, inflammatory bowel disease etc.). The Ethical Committee of “Bambino Gesù” Children's Hospital (No. OPBG-507 LB/2016) approved this study.

### 2.2. Methods

For each patients enrolled, demographic data (birth data, age, sex, race, and disease), anthropometric characteristics (BMI-z score and weight for age z-score) and symptoms (GERD, OPD, constipation and airway aspiration) were collected at the enrolment and at each follow-up visit. Data were collected at T0 (0–3 months after HEN beginning), T1 (2 years after HEN beginning) and T2 (the last follow up: time frame between 2 and 10 years). Data from T1 were analyzed only for nutritional status. Outcome measures for symptoms are described as the rate of patients with specific signs (clinical or instrumental) during the follow-up (T1 and T2).

#### 2.2.1. Comprehensive management

A multidisciplinary team including neurologists, therapists, speech therapists, gastroenterologists, endoscopists, surgeons, specialized nurses, dietitians, psychologists, pharmacists, and social workers has been involved in rehabilitation programs. As part of clinical management, in the following paragraphs, we describe the overall management methods concerning anthropometric assessment, GERD, constipation, Oropharyngeal Dysphagia (OPD), airway aspirations and EN management. All these aspects were investigated at the enrolment and during each follow-up visit.

##### 2.2.1.1. Anthropometric assessment

Patients' weight is assessed at any follow-up visit by wheelchair scales and sitting scales. Height has been measured as supine length or as knee-heel length in presence of joint contractions, spasticity, and/or scoliosis (6, 7). If wheelchair scales and sitting scales were unavailable, patients' weight has been assessed using the double weighing method (weight of the parent subtracted from combined weight of parent and child). Growth was assessed based on WHO growth charts up to 5 years, because these curves have been obtained in this range of age; in subjects older than 5 years the references are the CDC curves. Malnutrition has been diagnosed based on Body Mass Index (BMI) *z*-score and weight for age *z*-score, according to WHO and CDC growth charts (6, 8, 9). Malnutrition was classified, based on BMI *z*- score and age *z*-score, in mild (between −1 and −1.9 SD), moderate (between −2 and −2.9 SD) and severe (<-3 SD).

##### 2.2.1.2. GERD

**Figure 1:**
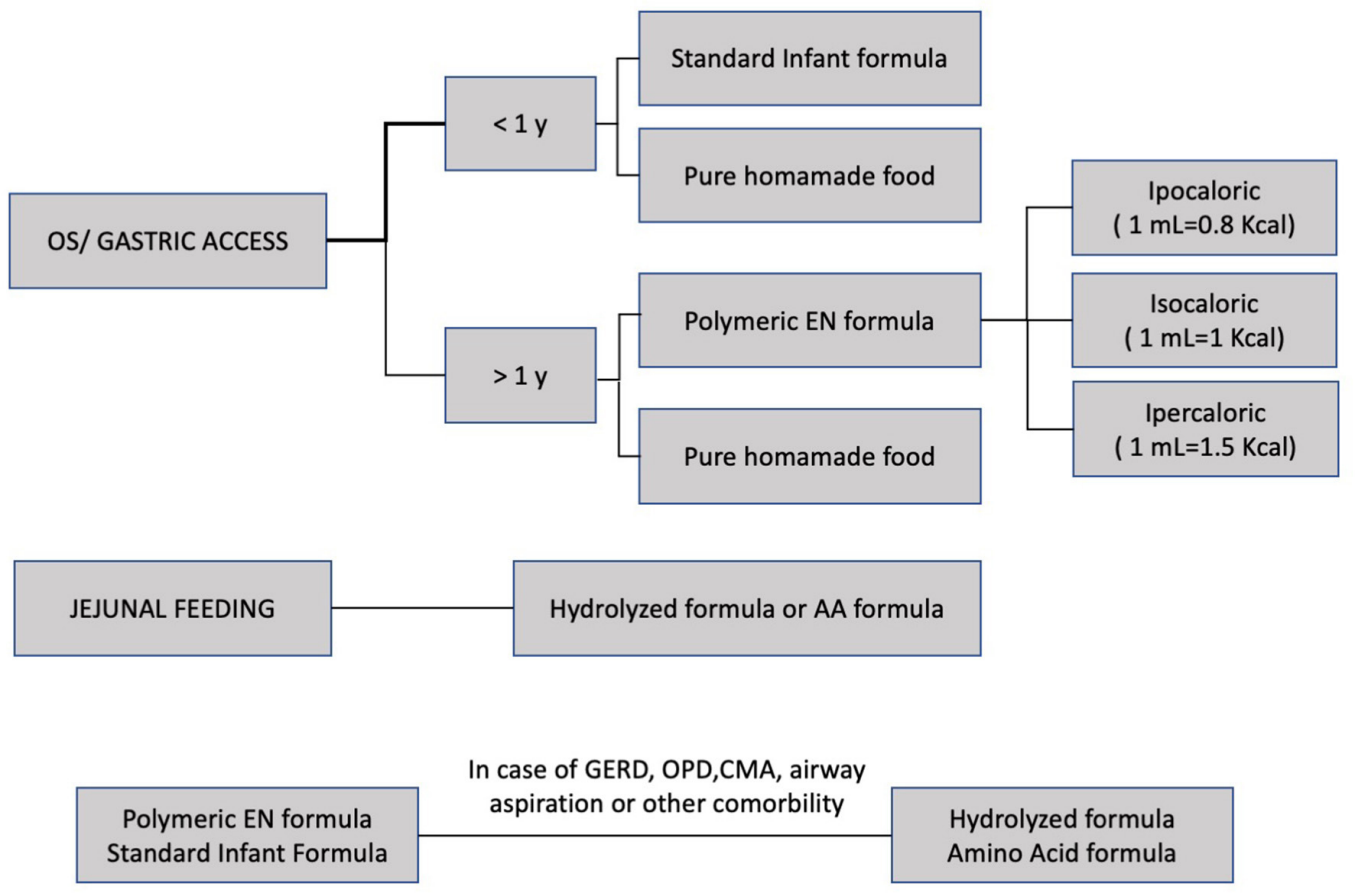
EN, enteral nutrition; GERD, gastroesophageal reflux disease; CMA, Cow's milk allergy.

GERD has been diagnosed based on partial or total remission of symptoms (vomiting, regurgitation, discomfort, unexplained pain, retching, and bloating) after proton pump inhibitor (PPI) treatment with or without abnormal findings of instrumental exams (esophago-gastro-duodenoscopy, gastric emptying-gastroesophageal reflux scintigraphy, upper gastrointestinal series, and 24-h esophageal pH-metry/pH-impedance). GERD has been treated with medical strategies, including long-term PPI (at standard and advised doses for age range) and EN modulation (Figure 1). Patients with non-responsive GERD (failure of overall medical strategies) have been undergoing Nissen fundoplication (NF) (2, 10–12).

##### 2.2.1.3. Constipation

Constipation was diagnosed based on clinical history (<3 stools per week) with or without suggestive imaging (abdominal radiograph). Medical management has been based on modulations of EN (Figure 1) and pharmacologic treatments (polyethylene glycol and periodic saline solution or sodium citrate and sorbitol enemas) (2, 13).

##### 2.2.1.4. OPD

The diagnosis of OPD has been established based on speech and language therapists, neurologists, gastroenterologists and pediatricians' clinical evaluation (altered sucking/swallowing, cough during meals, and/or mealtimes more than 4 h/day) and/or on the videofluoroscopy findings (2, 14).

##### 2.2.1.5. Airway aspirations

Airway aspirations were diagnosed based on clinical assessment, chest X-ray, image of aspiration from videofluoroscopy and/or radionuclide salivagram/gastric emptying-gastro esophageal reflux scintigraphy. Antegrade aspiration (evidence of airway aspiration from videofluoroscopy and/or from radionuclide salivagram) has been treated by discontinuation of oral feeding and starting of exclusive gastric EN; retrograde aspiration (evidence of airway aspiration from gastric emptying/gastro esophageal reflux scintigraphy) has been managed by switching gastric to jejunal feeding (2, 15).

#### 2.2.2. Enteral nutrition

According to guidelines (2), indications for EN are: inability to reach 60–80% of individual requirements for more than 10 days; total feeding time more than 4 h/day; inadequate growth or weight gain for more than 1 month according to the growth charts (8) in patients younger than 2 years; weight loss or no weight gain for more than 3 months in patients older than 2 years; change in weight for age over two growth channels on the growth charts and loss of at least 2 cm/year in height velocity rate compared with height velocity rate during the previous year in early/mid-puberty. EN programs started in Hospital and switched to HEN when EN duration was at least 8 weeks. For EN, different type of formula (Polymeric, hydrolyzed and aminoacidic formula) or mixed diet (EN formulas + ≤50% pureed homemade food) can be used.

Table 1 and Figure 1 summarize strategy to provide energy and fluids and to choose the enteral diet. Malnutrition and severe GERD have been treated with continuous EN or small bolus together with continuous EN; jejunal feeding was provided as continuous EN in every case (2, 4, 10–12, 16–29).

**Table 1:** Protocol for energy and fluid supply.

**Age**	Energy supply (lowest) ^*^	Energy supply (lowest)^*^	**Energy supply (highest)°**	**Energy supply (highest)°**	**Fluids supply**
	**M (kcal/Kg)**	**F (kcal/Kg)**	**M (kcal/die)**	**F (kcal/die)**	**M** = **F (ml/Kg)**
1–2 m	57/Kg	56.4/Kg	82.5/Kg	82.5/Kg	120–135/Kg
3–4 m	57/Kg	56.4/Kg	75/Kg	75/Kg	120–135/Kg
5–12 m	57/Kg	56.4/Kg	69/Kg	69/Kg	120–135/Kg
2 y	53.65/Kg	54.3/Kg	813.75	757.5	115–125/Kg
3 y	53.60/Kg	53/Kg	937.5	913.5	115–125/Kg
4 y	50.80/Kg	51/Kg	991.5	967.5	100–110/Kg
5 y	48.43/Kg	50.9/Kg	1,047.75	967.5	100–110/Kg
6 y	46.72/Kg	47.4/Kg	1,108.5	1,029	90–100/Kg
7 y	44.8/Kg	44.7/kg	1,179	1,096.5	90–100/Kg
8 y	41.5/Kg	42/Kg	1,259.25	1,166.25	90–100/Kg
9 y	40.3/Kg	39.1/Kg	1,349.25	1,432.5	90–100/Kg
10 y	38.3/Kg	37.1/Kg	1,658.25	1,505.25	70–85/Kg
11 y	36.6/Kg	35.2/Kg	1,758.75	1,593	70–85/Kg
12 y	35.1/Kg	32/Kg	1,874.25	1,689	70–85/Kg
13 y	33.4/Kg	30/kg	2,004	1,758	70–85/Kg
14 y	30.9/Kg	27/Kg	2,137.5	1,793.25	50–60/Kg
15 y	29.5/Kg	26/Kg	2,244.75	1,806.75	50–60/Kg
16 y	28.4/Kg	25.5/Kg	2,313.75	1,812	40–50/Kg
17 y	/	/	2,349	1,813.5	/
18 y	/	/	1,563.75	1,327.5	/

^*^This table reports Basal Metabolic Rate (lowest^*^ and highest° request for male and female) that should be adjusted for correction coefficients: tone factors (Hypertonia—multiply BMR by 1.1 (or adding 10%); Hypotonia—multiply BMR by 0.9 (or subtract 10%); then multiply the result for activity factors (Bedridden—multiply 1.15; Wheelchair dependent—multiply by 1.2; Crawling—multiply by 1.25; Ambulatory—multiply by 1.3) and finally add calories according to the actual growth (normal growth: add 31 kcal/day; undergrowth: add 150 kcal/day). At enteral nutrition starting we provide the amount of calories according to this equation.

* Energy supply is then modulated up to the values reported at the highest.

### 2.3. Statistical analysis

Data were expressed as medians and interquartile ranges (IQR) or frequencies. Differences in clinical variables were tested by the Fisher's exact test for categorical variables, the one-way ANOVA for normally distributed continuous variables and the Kruskal-Wallis's test for non-normally distributed continuous variables. The ANOVA was used for analysis of intention to treat during our follow-up, for anthropometric data. The Spearman's correlation coefficients were calculated to examine the univariable linear association of these variables with EN diet. Results have been adjusted for age, sex, severity (tetra-paresis, hemi- or other paresis), nutritional status, comorbidities, GI symptoms and presence of progressive or non-progressive neurological disease.

A *p*-value < 0.05 was considered statistically significant. Statistical analyzes were performed using Medcalc software, Version 20.014 (MedCalc Software Ltd., Ostend Belgium).

## 3. Results

Of 471 NI children enrolled, 45 (9.5%) patients were excluded (8 with co-existing digestive disorders; 18 on HEN before the first admission in our hospital; 15 with incomplete data and 4 on HPN) and 24 (5%) patients were lost at the follow-up. Therefore, 402 patients (M = 217, median age 39 months) were included. Median follow-up was 29 months. Tables 2, 3 report clinical characteristics of cohort and details of HEN, respectively.

**Table 2:** Summary of the main characteristics.

**No of patients**	**402**
**F/M**	46/54%
**Exitus**	3%
**Age at HEN beginning (months)**	39 (9-119)
**Follow up length (months)**	54 (29-66)
**Disease progression**	
NPNI patients	62%
PNI patients	38%
**PNI classification**	
Metabolic encephalopathies	31%
Neuromuscular diseases	22%
Leukoencephalopathy	16%
Genetic encephalopathy	14%
Malformative enecephalopathy	7%
West syndrome	5%
Rett syndrome	4%
Other	1%
**GMFCS**	
Level V	65%
Level IV	35%
**Nutritional status at baseline**	
Malnourished patients at baseline	70%
Not malnourished patients at baseline	30%

HEN, Home Enteral Nutrition; PNI, Progressive Neurologic Impairment; NPNI, Non-Progressive Neurologic Impairment; GMFCS, Gross Motor Function Classification System.

**Table 3:** Summary of HEN management.

	**T0**	**T2**	***P***
**Type of access route**			
*NGT*	22%	7%	< 0.0001
*PEG*	75%	85%	0.0003
PEG-J	3%	8%	NS
**Type of enteral diet**			
EN formula	53%	53%	NS
Mixed diet (HEN formula+ pureed homemade food covering ≤ 50% of the caloric intake)	21%	32%	0.0004
Standard infant formula	26%	15%	0.0001
**Type of HEN formulas**			
Polymeric formulas	63%	43%	< 0.0001
Hydrolyzed formulas	23%	38%	0.0098
Amino acid formulas	14%	19%	0.0096
**Mode of HEN delivery**			
Bolus	15%	32%	< 0.0001
Continuous + Bolus	66%	53%	0.0001
Continuous	19%	15%	0.0283

T0, at beginning; T2, at the last follow up; EN, Enteral Nutrition; HEN, Home Enteral Nutrition; NGT, Naso-Gastric Tube; PEG, Gastrostomy; PEG-J, Gastro-Jejunostomy.

### 3.1. Primary endpoint: HEN and outcomes

#### 3.1.1. Nutritional status

Nutritional status significantly improved after HEN beginning; growth profile significantly changed at T1. Table 4 shows a similar trend of weight for age *z*-score and BMI z-score. Patients on mixed diet showed a BMI *z-* score similar to the whole cohort (−0.9, IQ −2.2 0.2) at T2.

**Table 4:** Malnutrition and digestive co-morbidity after HEN beginning.

	**T0**	**T1**	**T2**
BMI (*z* score)	−2.4 (−5.2, −0.3)°	−1.4 (−3, 0.5)°	−0.8 (−2.8, 0.4)
Weight for age (*z* score)	−2.9 (−4.9, −0.9)^°°^	−1.9 (−3.7, −0.5)^°°^	−1.9 (−3.1, −0.3)
Malnutrition (%)	70^*^	/	44^*^
GERD (%)	74^§^	/	60^§^
Constipation (%)	43	/	44
OPD (%)	86	/	83
Airway aspiration (%)	17^∧^	/	6^∧^

BMI, body mass index; T0, at beginning; T1, 2 years after HEN beginning; T2, at the last follow up; GERD, Gastro esophageal Reflux Disease; OPD, Oropharyngeal dysphagia.

° p < 0.0001.

°° p = 0.0007.

* p = 0.0043.

§ p = 0.0003.

∧ p < 0.0001.

#### 3.1.2. GERD

GERD was diagnosed according to PPI response: clinical diagnosis was supported in 145 patients by esophago-gastro-duodenoscopy; in 181 by gastric emptying/gastro esophageal reflux scintigraphy; in 17 by upper gastrointestinal series and in 84 by 24-h esophageal pH-metry/pH-impedance study. The number of patients needing PPI at T2 (*n* = 242) was significantly lower than T1 (*n* = 299) (*p* = 0.0003).

#### 3.1.3. Constipation

Clinical diagnosis of constipation was proved by abdominal radiograph in 51 patients. No changes in constipation rate were observed over the study-period.

#### 3.1.4. OPD

The diagnosis of OPD was based on clinical assessment; 85 patients underwent videofluoroscopy. No significant changes in OPD rate were observed over the study-period.

#### 3.1.5. Airway aspirations

Airway aspirations significantly decreased after HEN beginning. We documented 67 episodes of airway aspirations (35 antegrade and 32 retrograde) at T0 and 16 episodes (13 antegrade and 3 retrograde) at T2.

### 3.2. EN diet and gastrointestinal complaints

**Figure 2:**
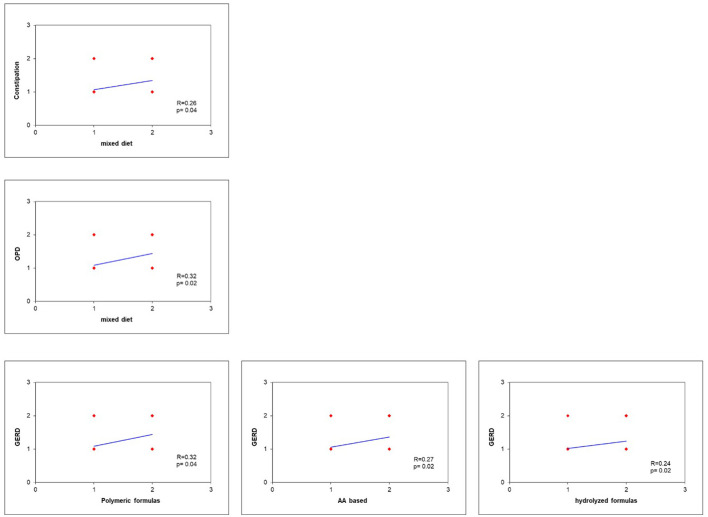
GERD results correlate with EN formula (*p* = 0.03), but not with mixed diet (*p* = 0.05). Polymeric formulas showed the highest correlation with GERD (*R* = 0.32), followed by AA based (*R* = 0.27) and hydrolyzed formulas (*R* = 0.24). Constipation and OPD were significantly correlated with mixed diet (*p* = 0.04, *R* = 0.26, and *p* = 0.02, *R* = 0.32, respectively).

GERD results correlate with EN formula (*p* = 0.03), but not with mixed diet (*p* = 0.05). Polymeric formulas showed the highest correlation with GERD (*R* = 0.32), followed by AA based (*R* = 0.27) and hydrolyzed formulas (*R* = 0.24). Constipation and OPD were significantly correlated with mixed diet (*p* = 0.04, *R* = 0.26 and *p* = 0.02, *R* = 0.32, respectively) (Figure 2).

### 3.3. Surgery

The probability to undergo Nissen significantly dropped after starting HEN (54 before HEN vs. 34 after HEN, *p* = 0.0314). Overall, 84 patients (21%) underwent Nissen; at baseline, they were significantly older than patients treated with HEN alone (median age 63 vs. 26 months, *p* = 0.02026); they had similar malnutrition rate (74 vs. 69%) but significantly higher GERD (100 vs. 68%, *p* < 0.0001), OPD (93 vs. 72%, *p* < 0.0001) and airway aspirations rate (29 vs. 13%, *p* < 0.0001). At the most recent follow up, GERD rate significantly decreased in patients who underwent Nissen compared to those who underwent HEN alone (21 vs. 62% respectively, *p* < 0.0001); OPD rate was similar (88 vs. 81% respectively, *p* NS) and airway aspirations rate were not significantly higher in patients who underwent Nissen than in those treated with HEN alone (12 vs. 5% respectively, *p* = 0.06). These results are reported in Table 5.

**Table 5:** Prevalence of clinical outcomes according to surgery and type of NI.

	**Malnutrition (%)**		**GERD (%)**		**Constipation (%)**		**OPD (%)**		**Airway aspirations (%)**	
	**T0**	**T2**	**T0**	**T2**	**T0**	**T2**	**T0**	**T2**	**T0**	**T2**
**Surgery yes/not**										
HEN+Nissen	74	42	100^§^	21^§^	45	48	93^§^	88	29^§^	12
HEN alone	69	46	68^§^	62^§^	43	42	72^§^	81	13^§^	5
**Type of NI disorder**										
NPNI	64	40	71	57	43	42	81	79	16	6
PNI	74	48	75	62	44	46	90	87	19	6

HEN, home enteral nutrition; NI, neurologic impairment; NPNI, non-progressive neurologic impairment; PNI, progressive neurologic impairment; GERD, Gastro-esophageal reflux disease; OPD, Oropharingeal dysphagia.

§ p < 0.0001 (by Fischer' exact test).

### 3.4. Role of neurological impairment

We also assessed whether different kinds of neurological impairment (PNI compared to NPNI) would affect the different clinical outcomes, i.e., GERD, constipation, OPD and airway aspiration rates. The statistical analysis, as shown in Table 5, failed to identify any statistically significant difference.

## 4. Discussion

Children with NI are an increasing subgroup of patients with medical complexity with feeding difficulties, malnutrition, and gastrointestinal disorders as distinctive features (1). In this study, we described growth and digestive co-morbidity in a large retrospective cohort of patients with NI following the beginning of HEN and the multidisciplinary management.

We managed HEN according to our Institutional protocol that was standardized and that took into consideration the advances in the most frequent and specific digestive co-morbidity of NI children.

We found that HEN results were effective in improving growth; malnutrition rate was 70% at T0 and decreased at 44% at T2. Our results confirmed data previously shown by Rosen et al. (30), that showed a significant catch-up growth after 6–12 of HEN in a small group of NI patients.

The high rate of malnutrition at baseline could be due to the high degree of functional damage (Gross Motor Function System Level V 65% and Level IV 35%) (31). Malnutrition may affect different outcomes such as risk of pneumonia, pressure ulcers, and pathological bone fractures as reported by Leonard et al. (32). Interestingly, in the present study, patients were significantly less malnourished 2 years after HEN beginning (T1), as shown by median BMI *z-*score that was −2.4 at T0 and −1.4 at T1.

Malnutrition was classified according to BMI *z-*score and to weight for age *z-*score but we did not include measures of triceps skinfolds and arm circumference because data were available only for few patients. The use of triceps skinfold to measure body composition should be a relevant part of the anthropometric investigations in patients with NI. Our study started more than 10 years ago and we used the defined malnutrition as BMI *z*-score, according to previous recommendations (6, 8, 9) but also as weight for age *z-*score, as indicated by the current guidelines (2). Last guidelines, indeed, recognize weight *z*-score and faltering weight and/or failure to thrive as red flags of malnutrition. From these premises, our way of assessing malnutrition, in spite of the retrospective nature of our study, can be considered consistent with the current ESPGHAN recommendations (2).

Furthermore, we found a rate of GERD of 74%, according to previous data (33), and of airways aspirations of 17% at T0, which decreased at 60 and 6%, respectively, at T2.

It is known that tube feeding accelerates gastric emptying compared to oral feeding (34, 35). Furthermore, HEN could improve GERD due to the increasing of liquid components (36) and due to the small meal size; these elements, together, optimize gastric emptying in severe GERD (30). On the other hand, thickening foods is considered a useful strategy to decrease and improve most of the reflux episodes (2). Our study confirms these data regarding the benefits of thickening foods on GERD: indeed, we found that EN formulas alone, but not mixed diet, were involved in GERD development. Moreover, based on our results, we hypothesize that hydrolyzed formulas had the lowest correlation with GERD, probably due to their attitude to fast gastric emptying (37–40).

During the study-period, 84 patients underwent NF; this surgery was effective in reducing GERD rate but not in preventing airway aspirations. NF was performed in children with severe GERD but 21% of this group had still GERD at T2; persistence of symptoms could be due to incomplete control of GERD. Our data confirmed data from Fukahori (41); in this study, multichannel intraluminal impedance was performed before and after NF surgery and differences were not found during the follow-up. Jejunal feeding also did not completely prevent GERD (41, 42).

During the study-period, type and dose of PPI was modulated based on sign and symptoms; indications to NF and GERD rate both decreased, showing that the multidisciplinary management including the new mode of feeding impacted on GERD rate. However, the retrospective nature of the study does not allow drawing clear conclusions on this aspect.

Airway aspirations rate decreases when oral feeding is replaced by a safer way of feeding (as nasogastric tube or PEG); based on these premises, HEN could be considered as an indirect strategy to prevent airway aspirations.

The most common comorbidity in the present series was OPD that affected 86% of the patients at T0 and 83% at T2, related likely to the severe impairment of gross motor dysfunction (3, 14, 43). Improvement of GERD could be responsible for the mild decrease of OPD during follow-up (37–40).

From literature (44, 45), prevalence of constipation was to 26% up to 74% in NI children; in our group, half of patients were constipated regardless of nutritional and pharmacological treatments. Constipation in this children is multifactorial; it is due to disease-specific factors (intestinal motility disorders, hypotonia, skeletal muscle discoordination, and skeletal deformities, combined with prolonged immobility); nutritional factors (low fiber and poor fluid intake) and pharmacological factors (e.g., anticholinergics and opiates) have negative effects on intestinal and colonic motility (46). Therefore, effective treatment of constipation in these patients could require combinations of drugs with different pharmacological action (47, 48). Regarding mixed diet, we planned to supply ≤50% of energy as pureed homemade foods; this approach is an emerging alternative to exclusive EN formulas (49, 50), but to date it is not officially approved (2).

Mixed diets were proposed to families who required this approach and to well-fed children carefully monitoring them to avoid nutritional deficiencies. In these families, sharing mealtime means sharing an important aspect of normal life, and for these reasons, we decide to satisfy their expectations. Moreover, patients on mixed diet showed at T2 a similar BMI *z-*score and lower GERD rate.

Main limitation of this study was the retrospective nature that made it descriptive and did not allow strictly associating HEN with outcomes. We considered primary endpoint modifications in nutritional status, because it is more likely dependent on changes in way of feeding, despite retrospectively assessment. The trend of further co-morbidity has been considered as a secondary endpoint that was related to the overall multidisciplinary management and not only to the feeding changes. However, the protocol for HEN management in our Institution was quite homogeneous and therefore highly reproducible in several clinical settings. We provided indeed a safe and effective nutritional protocol to address the nutritional approach in NI children and that represents the main strength of the present study. NI children could present poor growth and malnutrition due not only to feeding difficulties but also to factors related to underlying conditions (2, 31). The intrinsic attitude to poor growth makes it very difficult to estimate the energy needs of these children. Various methods have been proposed (21–23), but accurate estimation is difficult due to variations in energy requirements related to the heterogeneity of groups, altered body composition, and reduced physical activity levels. In such a perspective, our protocol could be a useful tool to approach nutritional programs in such medical complexity.

Further limitations are the following: (1) definition of GERD almost based on the response to PPI treatment, though supported by several investigations; (2) lack of measures of triceps skinfolds; (3) large use of scintigraphy as diagnostic tool for GERD and airway aspirations, although the role of this diagnostic tool is not currently well defined; (4) T2 is a large time frame because it is the time of the last follow-up, between 2 years (minimum time for the enrolment) and 10 years; and (5) antropometric data at baseline (T0) are collected 0–3 months after HEN beginning.

In conclusion, malnutrition and digestive complications are distinctive features in NI children. Nutritional status improves after 2 years from the beginning of standardized nutritional programs. Overall, multidisciplinary care, including standardized HEN protocols, seems to influence GERD and airway aspirations, which decreased over time. It is possible that constipation and OPD, unchanged over time, are more dependent on underlying diseases than on overall treatments. Future research should be addressed to develop further protocols of nutritional intervention, which meets specific and age-related requirements. Objective investigations are also required to support the diagnosis of digestive co-morbidity in such medical complexity; in particular, a wider use of scintigraphy, less invasive than other investigations, deserves to be detected.

## Data availability statement

The raw data supporting the conclusions of this article will be made available by the authors, without undue reservation.

## Ethics statement

The studies involving human participants were reviewed and approved by Ethical Committee of Bambino Gesù Children's Hospital (No. OPBG-507 LB/2016). Written informed consent to participate in this study was provided by the participants' legal guardian/next of kin.

## Author contributions

AD and TeC conceptualized and designed the study, coordinated and supervised data collection, drafted the initial manuscript, and reviewed and revised the manuscript. CMT, GB, FL, and BM designed the data collection instruments, collected data, and carried out the initial analyses. AM conceptualized and designed the study, coordinated and supervised data collection, and designed and revised statistics. PD, TaC, FD, SS, RP, and AC supervised data collection instruments and supervised the correct definition of outcomes and subgroups. GM critically reviewed the manuscript for important intellectual content. All authors approved the final manuscript as submitted and agree to be accountable for all aspects of the work.
